# Exploring large language model applications in dance education from an educational psychology perspective

**DOI:** 10.3389/fpsyg.2026.1746070

**Published:** 2026-05-19

**Authors:** Lan Zhao, Qi Huang, Kai Zhou, Langping Teng, Pengfei Tang

**Affiliations:** 1 School of Music and Dance, Hunan Women's University, Changsha, Hunan, China; 2 School of Music and Dance, Hunan University of Science and Engineering, Yongzhou, Hunan, China; 3 School of Social Development, Hunan Women's University, Changsha, Hunan, China; 4 Graduate School, Hunan University of Science and Engineering, Yongzhou, Hunan, China

**Keywords:** adaptive learning, cognitive framework, dance education, educational psychology, large language models

## Abstract

**Introduction:**

This study proposes a psychology informed framework for exploring the application of large language models (LLMs) in dance education. To address the limited personalization of traditional dance instruction and its insufficient adaption to learners' cognitive and emotional characteristics, a conceptual methodology is introduced with three components: Preliminaries, the DanceGPT, and Adaptive Knowledge Integration Strategy (AKIS).

**Methods:**

Within this framework, DanceGPT is designed as a multimodal architecture that integrates visual, motion, and textual information through multimodal encoding, learner aware personalization, Dance-Aware Attention, and an Adaptive Knowledge Integration Strategy (AKIS). From a system perspective, the current implemented and evaluated core is a multimodal video based action quality assessment model, in which computer vision and pose derived motion signals are fused to predict expert referenced performance scores, while the LLM-oriented tutoring and feedback functions remain part of the broader pedagogical framework. Experiments are conducted on the AQA-7 and FineDiving benchmarks under a fully supervised offline action quality regression setting.

**Results and discussion:**

Results show that the proposed model achieves consistent improvements over competitive baselines in ranking correlation and regression accuracy, demonstrating the effectiveness of multimodal fusion, domain guided attention, learner aware conditioning, and adaptive knowledge integration for dance related performance evaluation. Educational psychology is incorporated in this study primarily as a theoretical design lens for personalization and adaptive feedback rather than as a directly validated outcome dimension. The present work does not directly measure learning outcomes, tutoring effectiveness, or long term cognitive and emotional development. The reliance on multimodal modeling introduces non-trivial computational cost. These limitations indicate that future research should further validate the framework through real learner studies, interactive tutoring scenarios, and longitudinal evaluations in authentic dance education settings.

## 1 Introduction

Dance education serves as a cornerstone for cultural and artistic development, fostering creativity, emotional expression, and physical coordination. Despite its significance, traditional methods in dance education often struggle to meet the diverse needs of learners, provide personalized feedback, and incorporate psychological principles to optimize learning outcomes (Andersson, 2014). The integration of artificial intelligence (AI) into this domain offers transformative possibilities, enabling innovative teaching methodologies and bridging the gap between educational psychology and dance pedagogy (Gamble and Johnson, 2025). By utilizing AI technologies, educators can gain deeper insights into cognitive and emotional processes, enhance learning experiences, and develop adaptive systems tailored to individual learners. Moreover, the application of large language models (LLMs) in dance education opens new avenues for personalized content creation, effective communication, and increased engagement. This evolution is not merely technological but essential for addressing the limitations of traditional approaches, ensuring that dance education remains inclusive, accessible, and effective in the modern era (Papp-Danka and Oláh, 2021).

Early explorations into AI-based dance education primarily focused on structured systems that imitated human teaching logic through pre-defined instructional patterns (Wenn et al., 2018). These frameworks aimed to guide learners by replicating choreographic rules and movement hierarchies designed by experts. Although they provided an initial foundation for digitizing dance instruction, these systems were limited by their rigidity and lack of sensitivity to learners' cognitive diversity and emotional states. As a result, they often failed to accommodate the expressive, improvisational, and deeply human aspects of dance learning, which depend on adaptability and context-awareness (Li and Liang, 2024). Subsequent research advanced toward more flexible computational models capable of observing and interpreting dance practices directly from visual and motion-based data. Instead of relying solely on pre-defined structures, these models extracted meaningful features from real performances and learning sessions to offer feedback and improvement suggestions (Zhong et al., 2022). This shift enabled greater responsiveness to individual learning trajectories and performance variability. However, the effectiveness of these systems remained constrained by data limitations and their inability to fully interpret psychological factors such as motivation, attention, and emotional resonance, which are essential in the learning process (Yang and Li, 2022).

Recent developments in intelligent modeling have transformed this landscape through systems that combine perception, reasoning, and adaptive learning capabilities. Advanced neural architectures now allow AI tools to analyze intricate temporal patterns in dance, predict learner needs, and deliver emotionally intelligent feedback (Andersson, 2016). The introduction of large language models has further expanded these possibilities by generating personalized learning materials and contextually aware dialogues between instructors and learners. Despite these breakthroughs, significant challenges persist, including the need for interpretable models, equitable access, and alignment with educational psychology principles to ensure that technological innovation enhances, rather than replaces, the human and artistic essence of dance education (Connell, 2009).

Artificial intelligence (AI) has undergone rapid development in recent years, evolving from task specific algorithms toward more adaptive and general computational frameworks. Early studies have explored the conceptual foundations and optimization mechanisms of AI systems, including metaheuristic algorithms for complex problem solving (Aribowo, 2026). Recent work has further examined broader perspectives on machine intelligence and its evolution beyond human centered cognition, highlighting the expanding role of AI in future technological systems (Hamam, 2025). AI-driven optimization and control methods have been widely applied in engineering domains such as smart grids and robotics, demonstrating their effectiveness in handling dynamic and high dimensional environments (Aribowo et al., 2025). In the context of education, AI has been progressively applied to enhance learning support, automate assessment, and provide personalized feedback. However, most existing approaches are primarily designed for text based or structured learning scenarios. Extending AI techniques to skill based and embodied domains, such as dance education, remains a challenging problem due to the need for multimodal perception and fine grained motion understanding.

In dance education, effective instruction depends not only on accurate movement demonstration and performance evaluation, but also on how pedagogical support is adapted to learners' cognitive load, developmental stage, and emotional condition. From this perspective, educational psychology provides a useful foundation for designing intelligent dance learning systems, as principles such as cognitive load theory, scaffolding, and the zone of proximal development help explain why feedback should be selective, progressive, and learner sensitive rather than uniform across learners. Motivated by the limitations of symbolic AI, data driven methods, and conventional deep learning approaches, this study proposes a psychology informed multimodal framework for dance education that connects large language model (LLM)-oriented pedagogical design with video based action quality assessment. The framework takes multimodal inputs, including textual instructional information, visual video content, and pose derived motion representations, and integrates them through modality specific encoding, cross modal fusion, learner aware personalization, and adaptive knowledge integration. At the conceptual level, it is designed to support pedagogically relevant outputs such as movement analysis, feedback generation, and personalized instructional guidance; however, in the current implementation and evaluation, the core system is instantiated as a multimodal action quality assessment model that predicts expert referenced performance scores from single performer video clips. Within this design, educational psychology constructs are operationalized as model side inductive principles rather than directly measured outcome variables: learner related cognitive and emotional factors are incorporated as conditioning signals for personalization and adaptive knowledge integration, while pedagogical knowledge, psychological modulation, and domain specific guidance are integrated through the Adaptive Knowledge Integration Strategy. Accordingly, cognitive load, scaffolding, and learner developmental considerations function here as design principles for adaptive representation learning and feedback conditioning rather than as experimentally verified learner outcomes. The main contribution of this study therefore lies in the development and technical validation of a psychology informed multimodal framework for dance related performance evaluation. Empirical validation is conducted under an offline supervised action quality assessment setting on benchmark datasets, where the primary objective is to evaluate prediction accuracy rather than to directly measure learning outcomes, motivation, emotional engagement, or classroom experience. The broader pedagogical effectiveness of the framework remains to be examined in future learner centered and longitudinal studies.

The main contributions of this study are summarized as follows:

- A psychology informed multimodal framework for dance education is proposed, which integrates textual, visual, and motion related inputs through learner aware personalization, domain guided attention, and adaptive knowledge integration.
- The current implemented system is formulated and evaluated as a supervised action quality assessment model that predicts continuous expert referenced performance scores from dance related action videos, providing a technical proxy for performance evaluation in educational settings.
- Educational psychology is operationalized through model mechanisms rather than direct human subject outcome measurement, including cognitive emotional conditioning, pedagogical knowledge integration, and adaptive modulation within the proposed framework; accordingly, the present empirical study focuses on technical validation, while pedagogical impact remains future work.

Existing studies on AI in dance education have primarily focused on motion analysis, pose estimation, and performance evaluation using computer vision techniques. These approaches have achieved notable progress in action recognition and quality assessment, providing objective measures of movement performance. Recent advances in large language models have enabled new forms of instructional support, such as automated tutoring and feedback generation in text based educational settings. However, several important gaps remain. First, most motion based approaches treat performance assessment as an isolated prediction task, without explicitly connecting the learned representations to pedagogical objectives or instructional feedback. Second, existing LLM-based educational systems are largely designed for text centric domains and do not incorporate multimodal motion information, limiting their applicability to skill based disciplines such as dance. Third, there is a lack of unified frameworks that integrate motion understanding, domain knowledge, and pedagogically relevant constraints within a single modeling pipeline. To address these limitations, this work proposes a multimodal framework centered on DanceGPT and the Adaptive Knowledge Integration Strategy (AKIS). The proposed approach bridges motion representation learning with instruction aware modeling by incorporating domain knowledge and pedagogical constraints into the learning process, thereby providing a structured foundation for future feedback generation and educational applications.

## 2 Related work

Existing studies related to this work can be broadly grouped into three strands: dance education and assessment, technology-enhanced and AI-supported dance learning, and adaptive learning with large language models in education. In the present study, computer vision is treated as a supporting module for extracting movement-related information rather than as the conceptual core of the framework.

### 2.1 Dance education and assessment

Research in dance education has increasingly emphasized that dance should not be understood merely as technical skill training, but also as a pedagogically rich domain involving creativity, reflective practice, teacher preparation, and curriculum design. Dance education has been discussed as a transdisciplinary vehicle for transforming teacher education and promoting broader academic development (Cook, 2025). Related curriculum reform studies have further highlighted the value of outcome-based and learner-centered approaches in cultivating students' creativity and social adaptability (Jianping and Chin, 2024). At the same time, assessment-oriented research has shown that effective dance teaching depends not only on demonstration and correction, but also on pedagogically grounded evaluation practices. Dance teachers' experiences of assessment reveal the importance of aligning instructional support with learners' developmental prerequisites (Andersson, 2018), while research on teacher preparation has stressed the value of shared criteria and consensus-based evaluation in dance education (Schmid, 2019). These studies suggest that intelligent systems for dance education should be situated within a broader educational context that includes teaching practice, assessment, and learner development.

### 2.2 Technology-enhanced and AI-supported dance learning

A related line of work concerns the integration of technology into movement and dance education. Earlier studies pointed out that digital technologies could support dance learning through new forms of visualization, communication, and skill development (Dania et al., 2011). Subsequent research explored smart technologies for supporting dance education (Dias Pereira dos Santos, 2017), as well as artificial intelligence applications for learners with special educational needs (Hu and Wang, 2021). More recent studies have reported customized online learning approaches for dance majors (Li et al., 2024) and extended-reality interactive systems for dance training (Xu et al., 2023), indicating that technology-enhanced dance education is gradually moving toward more personalized, interactive, and multimodal learning environments. These developments provide important context for the present work. However, unlike prior studies that mainly focus on technology adoption or digital teaching practice, the present study is concerned with how multimodal computational models can be aligned with pedagogical and psychological considerations in dance-related performance evaluation.

### 2.3 Adaptive learning and large language models in education

Another important foundation comes from adaptive learning and intelligent tutoring systems. Existing research has established that learner modeling, instructional adaptation, and individualized support are central to effective educational technologies. Recent systematic review evidence has further shown that intelligent tutoring systems in authentic educational settings are increasingly expected to remain sensitive to learner diversity and real instructional practice (Wang et al., 2023). This perspective is especially relevant to dance education, where students differ substantially in prior experience, motor ability, pacing, and feedback needs. In parallel, large language models have expanded the scope of AI-supported education by enabling conversational tutoring, instructional assistance, adaptive guidance, and educational question answering. Emerging work has discussed LLM-based personalized education in conjunction with adaptive learning (Wen et al., 2024), explored knowledge-graph-supported educational question-answering systems (Bui et al., 2024), and presented case studies on the development of LLM-based educational tools in practice (Van Campenhout et al., 2025). These studies indicate that LLMs offer promising opportunities for connecting multimodal evidence with pedagogically meaningful communication, while also motivating careful task grounding in real educational contexts.

## 3 Method

### 3.1 Overview

This section provides a detailed examination of the integration of large language models (LLMs) into dance education, framed within the context of educational psychology. The primary objective of this study is to explore the potential of LLMs to enhance learning processes, refine pedagogical methodologies, and address domain-specific challenges inherent to dance education. To systematically approach this objective, the methodology is structured into three core components: *Preliminaries, Cognitive-Informed Generative Framework*, and *Adaptive Pedagogical Strategy*.

In Section 3.2, the foundational aspects of incorporating LLMs into dance education are established. This includes defining the essential elements of dance education, encompassing movement acquisition, choreography comprehension, and emotional articulation, and translating these elements into computational representations compatible with LLMs. The theoretical basis for this integration is grounded in principles from educational psychology, including cognitive load theory, scaffolding, and the zone of proximal development. These principles inform the design and implementation of the proposed framework and strategy, ensuring alignment with established pedagogical theories. In Section 3.3, the *DanceGPT* is introduced as a novel approach to utilizing LLMs for generating personalized and context-sensitive educational content tailored to dance learners. The framework's architecture is described in detail, encompassing mechanisms for processing and generating dance-related information, including movement instructions, choreography guidance, and evaluative feedback. The DanceGPT is explicitly designed to address the cognitive and emotional dimensions of dance learning, leveraging the theoretical insights outlined in the preliminaries to optimize its functionality and relevance. In Section 3.4, the *Adaptive Knowledge Integration Strategy (AKIS)* is presented as a practical implementation methodology for deploying the DanceGPT in real-world dance education scenarios. This strategy emphasizes the dynamic interaction between learners and the LLM, ensuring that the generated content is responsive to individual skill levels, learning styles, and emotional conditions. The AKIS incorporates mechanisms such as iterative feedback loops, adaptive learning trajectories, and multimodal engagement to foster a comprehensive and effective educational experience. The design and evaluation of AKIS are deeply informed by educational psychology, ensuring its alignment with the cognitive and emotional development needs of learners. This structured methodology aims to bridge the gap between advanced computational technologies and the pedagogical requirements of dance education, offering innovative solutions for educators and learners. By integrating theoretical foundations with practical applications, the study contributes to the development of tools that enhance the educational landscape of dance through the capabilities of LLMs.

**Figure 1:**
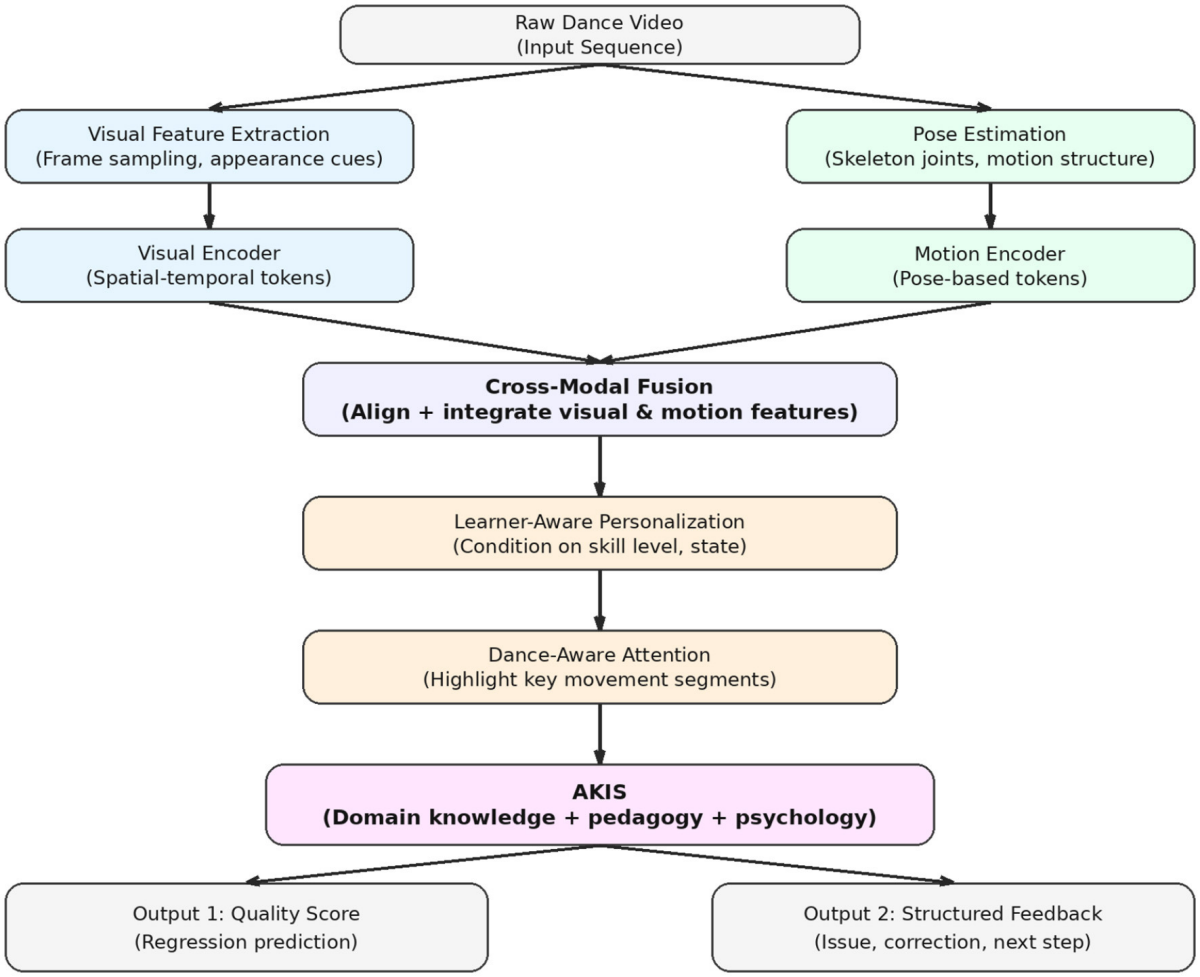
End-to-end pipeline of the proposed framework. A raw dance video is processed through a visual branch (appearance features) and a pose branch (skeletal motion). The encoded features are fused into a multimodal representation, followed by learner-aware personalization and Dance-Aware Attention. The Adaptive Knowledge Integration Strategy (AKIS) further incorporates domain knowledge and pedagogical constraints. The framework supports both quality score prediction (validated in experiments) and structured pedagogical feedback generation.

Figure 1 illustrates the end-to-end pipeline from visual input to pedagogical output. The process starts with a raw dance video, which is processed through two parallel branches: a visual branch for appearance based features and a pose branch for skeletal motion representation. These features are encoded into latent tokens and integrated through cross modal fusion to form a unified multimodal representation. The fused representation is further conditioned by learner aware personalization and refined through Dance-Aware Attention to highlight pedagogically relevant motion segments. It is then passed to the Adaptive Knowledge Integration Strategy (AKIS), where domain knowledge, pedagogical constraints, and psychological factors are incorporated. The final output supports two forms: a continuous quality score for action assessment, which is used in the current experiments, and structured feedback, such as identifying key issues and providing corrective guidance, as part of the broader pedagogical framework.

### 3.2 Preliminaries

In this subsection, we formalize the problem of exploring large language model (LLM) applications in dance education from an educational psychology perspective. The goal is to establish a mathematical framework that encapsulates the interaction between LLMs, dance pedagogy, and psychological principles, enabling a structured approach to analyze and optimize their integration.

Let D represent the domain of dance education, which includes a set of pedagogical tasks $T=\left\{T_{1},T_{2},\ldots ,T_{n}\right\}$, where each task *T*_*i*_ corresponds to a specific educational activity, such as choreography generation, movement analysis, or feedback provision. Each task *T*_*i*_ is associated with a set of input features ${\text{x}}_{i}\in {\mathbb{R}}^{d}$, where *d* is the dimensionality of the feature space, and an output ${\text{y}}_{i}\in {\mathbb{R}}^{k}$, where *k* represents the dimensionality of the output space.

The interaction between LLMs and dance education can be modeled as a mapping function *f*:ℝ^*d*^ → ℝ^*k*^, parameterized by θ, such that Equation 1:


$$\begin{array}{l} {\text{y}}_{i}=f\left({\text{x}}_{i};\theta \right), \end{array}$$ (1)

where θ represents the parameters of the LLM. The function *f* is designed to capture the relationship between input features **x**_*i*_ and the desired outputs **y**_*i*_, which may include textual explanations, movement suggestions, or psychological insights.

To incorporate educational psychology principles, we define a set of psychological factors $P=\left\{P_{1},P_{2},\ldots ,P_{m}\right\}$, where each factor *P*_*j*_ represents a specific psychological construct, such as motivation, cognitive load, or emotional engagement. Each factor *P*_*j*_ is modeled as a function $g_{j}:{\mathbb{R}}^{d}\to \mathbb{R}$, such that Equation 2:


$$\begin{array}{l} P_{j}=g_{j}\left({\text{x}}_{i}\right), \end{array}$$ (2)

where *g*_*j*_ quantifies the impact of the input features **x**_*i*_ on the psychological construct *P*_*j*_. The overall psychological impact $P_{\text{total}}$ is then defined as Equation 3:


$$\begin{array}{l} P_{\text{total}}=\overset{m}{\underset{j=1}{\sum }}w_{j}P_{j}, \end{array}$$ (3)

where *w*_*j*_ represents the weight of each psychological factor in the overall impact.

The optimization problem can be formulated as finding the optimal parameters θ^*^ of the LLM that maximize the educational outcomes while considering psychological factors. This can be expressed as Equation 4:


$$\theta^{*}=\text{arg}\underset{\theta }{\text{max}}ℒ(y,\hat{y})-\text{λ}P_{\text{total}},$$ (4)

where $ℒ(y,\hat{y})$ is a loss function that measures the discrepancy between the predicted outputs $\hat{y}$ and the ground truth **y**, and λ is a regularization parameter that balances the trade-off between educational outcomes and psychological impact.

To further refine the model, we introduce a temporal component to account for the dynamic nature of dance education. Let *t* ∈ *T* represent discrete time steps, where *T* = {1, 2, …, *T*}. The input features **x**_*i*_ and outputs **y**_*i*_ are now time-dependent, denoted as **x**_*i*_(*t*) and **y**_*i*_(*t*), respectively. The mapping function *f* is extended to Equation 5:


$$\begin{array}{l} {\text{y}}_{i}\left(t\right)=f\left({\text{x}}_{i}\left(t\right);\theta \left(t\right)\right), \end{array}$$ (5)

where θ(*t*) represents the time-dependent parameters of the LLM. The psychological factors P are also time-dependent, denoted as *P*_*j*_(*t*), and the overall psychological impact becomes Equation 6:


$$\begin{array}{l} P_{\text{total}}\left(t\right)=\overset{m}{\underset{j=1}{\sum }}w_{j}P_{j}\left(t\right). \end{array}$$ (6)

The optimization problem is now formulated as Equation 7:


$$\theta^{*}(t)=\underset{\theta (t)}{\arg\max}\sum_{t=1}^{T}\left[ℒ(y(t),\hat{y}(t))-\text{λ}P_{\text{total}}(t)\right].$$ (7)

To ensure the robustness of the model, we introduce constraints that capture the inherent properties of dance education and psychological principles. For example, the outputs **y**_*i*_(*t*) must satisfy physical constraints related to human movement, denoted as $C_{\text{physical}}$, and psychological constraints related to cognitive and emotional states, denoted as $C_{\text{psychological}}$. These constraints can be expressed as Equation 8:


$$\begin{array}{l} {\text{y}}_{i}\left(t\right)\in C_{\text{physical}},\;P_{\text{total}}\left(t\right)\in C_{\text{psychological}}. \end{array}$$ (8)

The final optimization problem is Equation 9:


$$\theta^{*}(t)=\underset{\theta (t)}{\text{arg max}}\sum_{t=1}^{T}\left[ℒ(y(t),\hat{y}(t))-\text{λ}P_{\text{total}}(t)\right],$$ (9)

subject to Equation 10:


$$\begin{array}{l} {\text{y}}_{i}\left(t\right)\in C_{\text{physical}},\;P_{\text{total}}\left(t\right)\in C_{\text{psychological}}. \end{array}$$ (10)

### 3.3 DanceGPT: a novel framework for dance education

**Figure 2:**
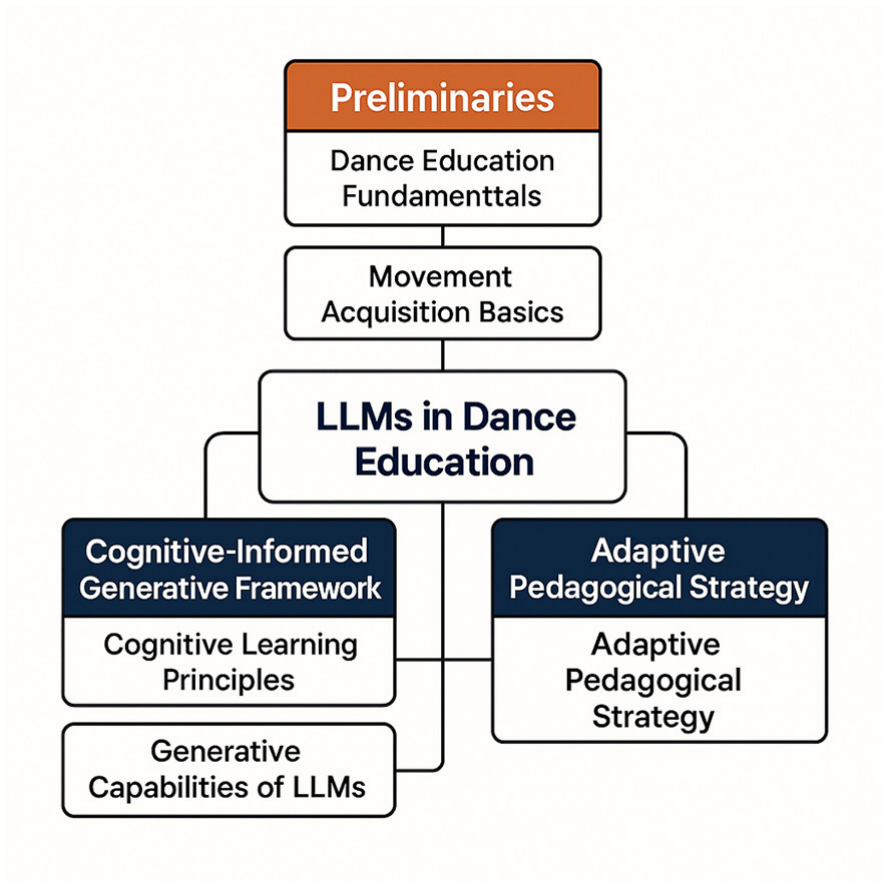
Conceptual framework for integrating large language models (LLMs) into dance education. The framework consists of three key components: Preliminaries, which define the fundamentals of dance education and movement acquisition; the Cognitive-Informed Generative Framework, which applies cognitive learning principles and LLMs' generative capabilities; and the Adaptive Pedagogical Strategy, which ensures responsive and personalized teaching through adaptive strategies.

In this subsection, we introduce our novel framework (As shown in Figure 2), DanceGPT, which leverages the capabilities of large language models (LLMs) to enhance dance education through an educational psychology perspective. DanceGPT is designed to address the unique challenges in teaching and learning dance by integrating domain-specific knowledge, personalized learning strategies, and interactive feedback mechanisms. The model is built upon a transformer-based architecture, incorporating innovative modifications to adapt to the pedagogical requirements of dance.

**Figure 3:**
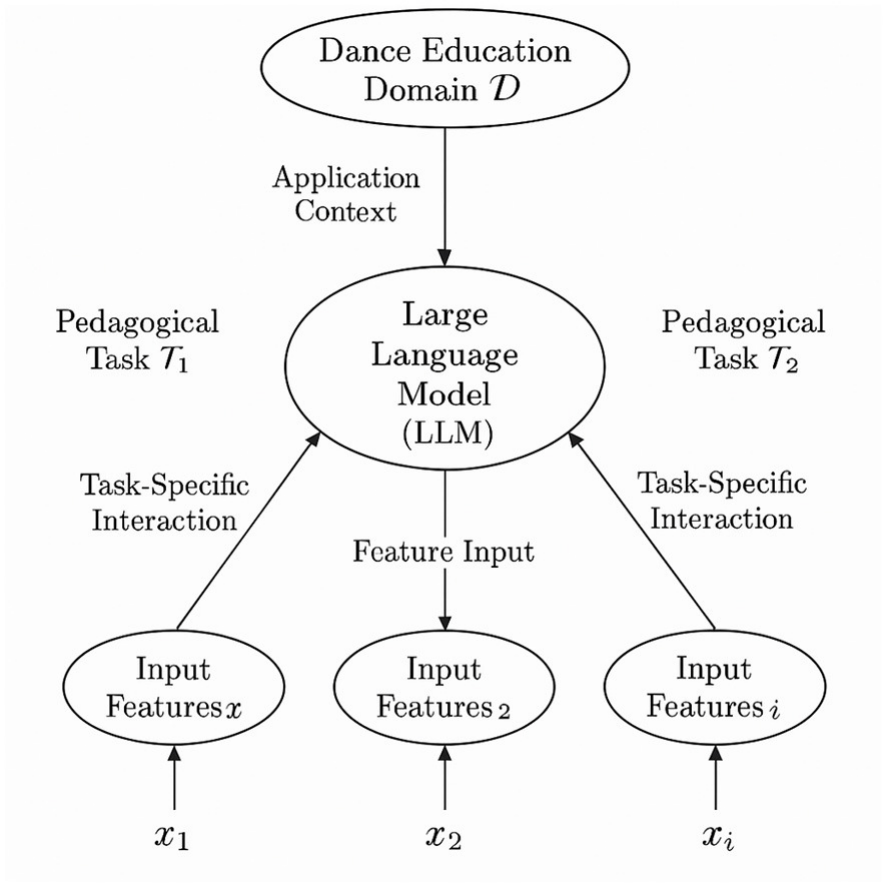
Schematic representation of the interaction between the dance education domain, pedagogical tasks, and a large language model (LLM). The LLM receives input features *x*_*i*_ corresponding to specific tasks $T_{i}$ and generates task-specific outputs. This structured interaction enables the LLM to perform diverse educational functions such as movement analysis, feedback generation, and choreography instruction.

**Multimodal Encoding Integration:** DanceGPT utilizes a structured multimodal input pipeline to represent diverse educational signals including textual instructions, visual content, and motion sequences (As shown in Figure 3). Each input modality is encoded through a dedicated encoder, yielding distinct embeddings, which are then concatenated and aligned in a shared representational space. The multimodal embedding **X** is computed as Equation 11:


$$\begin{array}{l} \text{X}={\text{X}}_{t}⊕{\text{X}}_{v}⊕{\text{X}}_{m}, \end{array}$$ (11)

where **X**_*t*_, **X**_*v*_, and **X**_*m*_ represent the textual, visual, and motion data respectively, and ⊕ denotes the concatenation operation. Each component is processed independently (Equation 12):


$$\begin{array}{l} {\text{E}}_{i}={\text{Encoder}}_{i}\left({\text{X}}_{i}\right),\;i\in \left\{t,v,m\right\}. \end{array}$$ (12)

These modality-specific embeddings are then integrated using cross-attention to form a comprehensive fused representation (Equation 13):


$$\begin{array}{l} {\text{E}}_{f}=\text{CrossAttention}\left({\text{E}}_{t},{\text{E}}_{v},{\text{E}}_{m}\right). \end{array}$$ (13)

**Learner-Aware Personalization:** To adapt to individual differences in learners, DanceGPT incorporates a personalization module that conditions the fused multimodal representation on learner-specific cognitive and emotional attributes. This adaptation ensures the outputs align with the learner's developmental stage and educational needs. Let L denote the vector of learner attributes. The personalized representation **E**_*p*_ is computed as Equation 14:


$$\begin{array}{l} {\text{E}}_{p}=P\left({\text{E}}_{f},L\right), \end{array}$$ (14)

where P is a learned transformation that models the interaction between educational content and learner profiles. The personalized embedding **E**_*p*_ is used as the input to the prediction decoder (Equation 15):


$$\begin{array}{l} \text{y}=D\left({\text{E}}_{p}\right), \end{array}$$ (15)

where **Y** represents a sequence of predicted dance instructions or feedback responses tailored to the learner.

**Dance-Aware Attention:** DanceGPT introduces a novel domain-specific attention mechanism, termed *Dance-Aware Attention* (DAA), which enhances the model's focus on pedagogically salient elements within the dance domain. The attention weights are guided by the structural hierarchy and pedagogical importance of dance movements. Let **E**_*p*_ be the personalized embedding, and D denote the domain knowledge base. The DAA mechanism computes the weights **A** as Equation 16:


$$\begin{array}{l} \text{A}=\text{Softmax}\left(\text{Q}{\text{K}}^{T}/\sqrt{d_{k}}\right), \end{array}$$ (16)

where **Q** and **K** are linear projections of **E**_*p*_ influenced by D. The attended representation is Equation 17:


$$\begin{array}{l} {\text{E}}_{a}=\text{A}\text{V}, \end{array}$$ (17)

and this representation is used by a feedback generator to produce actionable pedagogical suggestions (Equation 18):


$$\begin{array}{l} \text{F}=F\left({\text{E}}_{a},L\right), \end{array}$$ (18)

where **F** denotes the generated feedback, dynamically adapted to the learner's current progress and pedagogical context.

### 3.4 Adaptive knowledge integration strategy

**Figure 4:**
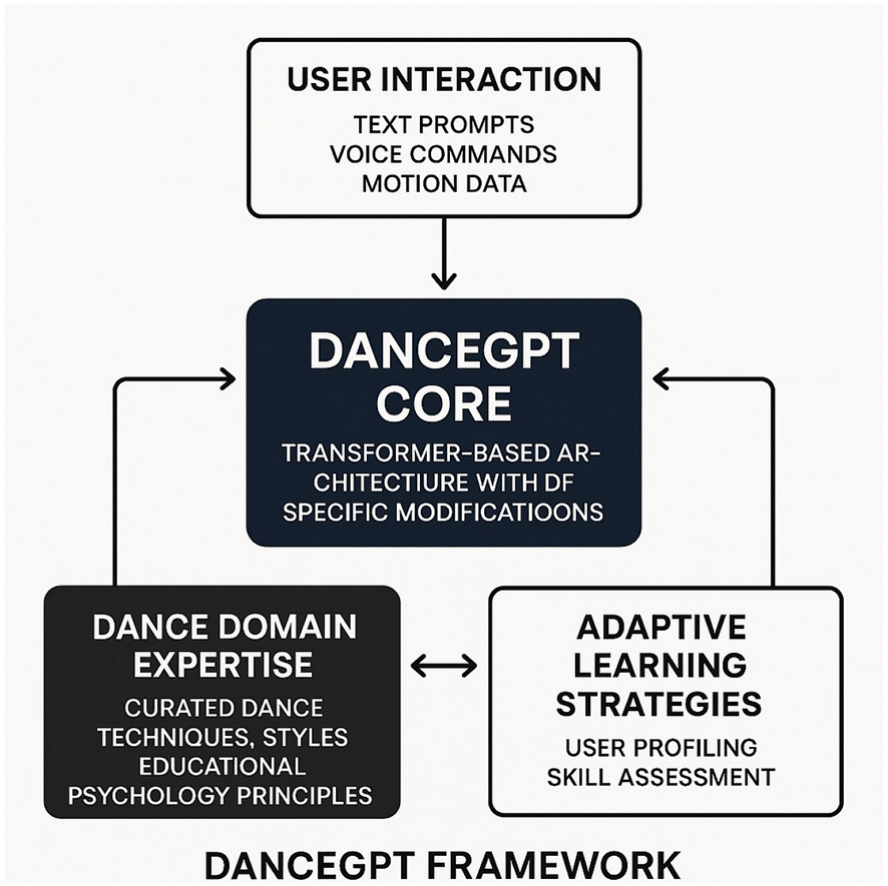
Architecture of the DanceGPT framework. User interactions are processed by the core transformer-based module, which is customized for dance education. The system integrates curated dance domain expertise and adaptive learning strategies (including user profiling and skill assessment) to provide responsive and personalized instruction.

In this subsection, we present the Adaptive Knowledge Integration Strategy (AKIS), a novel mechanism designed to enhance the adaptability of large language models (LLMs) in dance education(As shown in Figure 4). AKIS integrates domain knowledge, pedagogical reasoning, and psychological adaptation into a unified framework that dynamically adjusts to learners' cognitive and emotional states. The strategy is grounded in educational psychology and is engineered to ensure that model-generated guidance is both pedagogically effective and emotionally resonant. It establishes a structured interaction loop between the model, learner feedback, and domain-specific knowledge representations.

**Figure 5:**
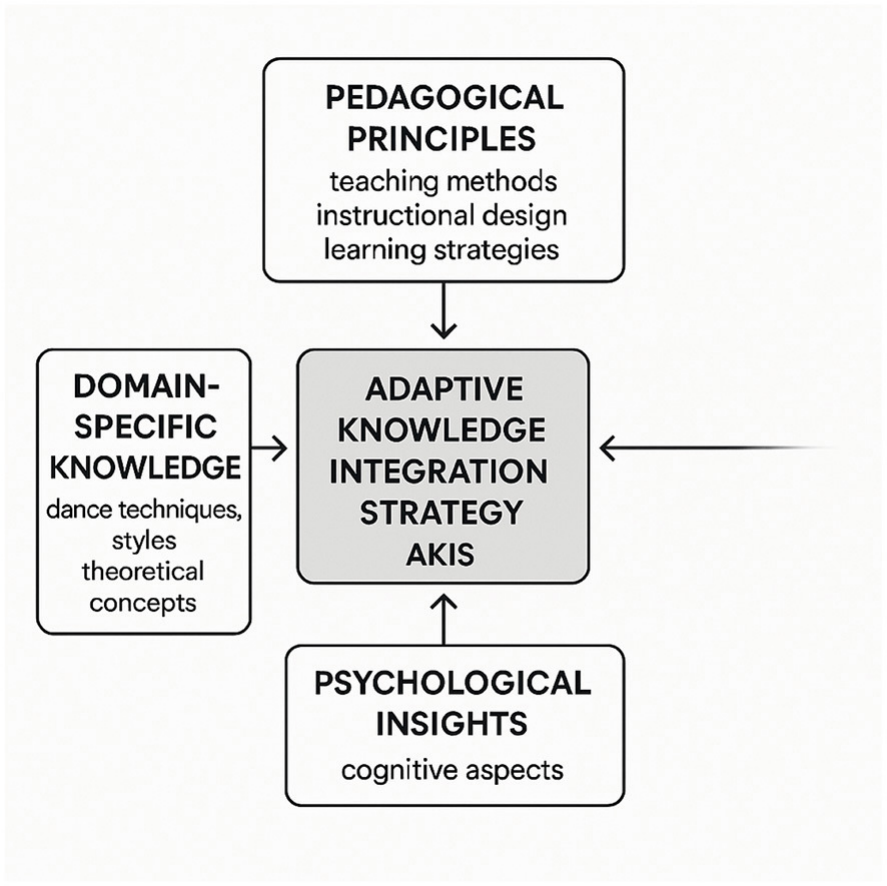
Conceptual overview of the Adaptive Knowledge Integration Strategy (AKIS). The framework synthesizes pedagogical principles, domain-specific knowledge, and psychological insights to deliver context-aware, personalized guidance in dance education.

**Dynamic Knowledge Fusion:** AKIS employs a dynamic fusion mechanism to integrate heterogeneous sources of knowledge including dance-specific movements, instructional cues, and psychological parameters into a coherent representational space(As shown in Figure 5). Let S denote the set of dance styles, P the set of pedagogical techniques, and E the set of emotional features. These elements are connected within a weighted knowledge graph G=(V,R,W), where V=S∪P∪E and **W** encodes the relational strength between nodes. The integrated representation **K** is computed via a diffusion-based fusion process (Equation 19):


$$\begin{array}{l} \text{K}=\sigma \left(\text{W}\text{V}{\text{W}}^{T}+\gamma \text{I}\right), \end{array}$$ (19)

where σ is an activation function, γ is a regularization coefficient, and **I** is the identity matrix. This process enables AKIS to infer latent pedagogical associations, aligning model reasoning with both domain structure and learner context.

Psychological State Modulation: AKIS adapts to each learner's psychological condition through a modulation mechanism that aligns generated content with individual emotional and cognitive states. Let **h**_*c*_ and **h**_*e*_ denote cognitive and emotional embeddings extracted from learner interactions. The model combines these dimensions using an adaptive weighting function that evolves with learning progression (Equation 20):


$$\begin{array}{l} {\text{z}}_{\psi }={\text{λ}}_{c}{\text{h}}_{c}+{\text{λ}}_{e}{\text{h}}_{e}, \end{array}$$ (20)

where λ_*c*_+λ_*e*_ = 1, and the weights λ_*c*_, λ_*e*_ are dynamically updated based on feedback signals from the learner model. The modulated psychological representation **z**_ψ_ is incorporated into the generative process through attention reweighting (Equation 21):


$$\begin{array}{l} {\text{A}}_{\psi }=\text{Softmax}\left(\frac{\text{Q}{\left(\text{K}+{\text{z}}_{\psi }\right)}^{T}}{\sqrt{d_{k}}}\right), \end{array}$$ (21)

allowing the model to emphasize emotionally supportive and cognitively appropriate responses within the instructional output.

**Feedback-Driven Adaptation:** To ensure continuous personalization, AKIS establishes a feedback optimization loop that updates the model parameters based on learner evaluations. Let Θ denote the model parameters and F represent the feedback mapping from learner response *r* to parameter updates. The adaptive update rule is formulated as Equation 22:


$$\begin{array}{l} {\text{Θ}}_{t+1}={\text{Θ}}_{t}+\eta \nabla_{\text{Θ}}F\left(r,q\right), \end{array}$$ (22)

where η is the adaptation rate, and $\nabla_{\text{Θ}}F\left(r,q\right)$ captures the gradient of the feedback function with respect to the parameters. The refined model state generates a recalibrated response (Equation 23):


$$\begin{array}{l} r^{\prime }=L_{{\text{Θ}}_{t+1}}\left(q\right), \end{array}$$ (23)

### 3.5 Prompting, output schema, and psychology-guided instruction rules

To make the pedagogical mechanism of the proposed framework more explicit, this subsection specifies how the LLM-oriented component can be operationalized through structured prompts, constrained output schemas, and psychology guided instructional rules. The purpose of this addition is to clarify the instructional interface of the framework and the manner in which educational psychology principles are translated into implementable guidance constraints. Cognitive load regulation, scaffolding, and learner sensitive adaptation are reflected not as unrestricted free form generation, but as structured control signals that shape the content density, instructional order, and personalization level of the generated response. Within this design, the prompt is treated as a structured carrier of learner information, task context, multimodal evidence, and pedagogical objectives. A prompt instance may include the learner level, current task, motion related evidence derived from video and pose representations, detected weaknesses, and the intended teaching objective. Instead of allowing unconstrained output, the framework uses a pre-defined schema to organize the response into interpretable fields, such as movement focus, primary issue, evidence basis, corrective cue, rehearsal target, and encouragement. This structured design improves pedagogical interpretability and makes the generated guidance easier to align with instructional goals in dance education. From the perspective of scaffolding, the prompting mechanism follows a progressive instructional logic in which diagnosis precedes correction, and correction precedes subsequent practice. The generated feedback is therefore constrained to begin with the most important issue, followed by a concrete action cue, and then by a manageable next step exercise. This design prevents the learner from being exposed to too many concurrent corrections and supports gradual skill acquisition. For novice learners, the prompt rules restrict the output to one dominant problem and one immediate correction target. For more advanced learners, the response can include an additional refinement cue or a short staged sequence, while still maintaining a clear priority order. From the perspective of cognitive load theory, the prompt rules explicitly regulate response complexity. The number of simultaneously presented corrections is limited, terminology is simplified for less experienced learners, and the length of each instructional unit is constrained to remain concise and actionable. In this way, the framework reduces extraneous load, preserves attention on the most pedagogically relevant movement feature, and avoids overwhelming the learner with excessive verbal explanation. The learner aware personalization module and the Adaptive Knowledge Integration Strategy further support this process by aligning feedback phrasing and emphasis with learner specific cognitive and emotional conditions. Prompting in the proposed framework is not viewed as an isolated text generation operation, but as a rule governed instructional interface grounded in pedagogical sequencing, domain knowledge, and psychological adaptation. Representative prompt templates, output schemas, and the corresponding scaffolding and cognitive load constraints are summarized in Table 1.

**Table 1:** Representative prompt templates and psychology-guided output constraints in the proposed framework.

Function	Prompt example	Output schema	Instruction rule
Movement analysis	Learner level: novice. Task: basic dance phrase. Based on motion evidence, identify the main problem and provide one correction.	Issue, cue, next step	Scaffolding: focus on one key problem first. Cognitive load: keep feedback short and concrete.
Formative feedback	Learner level: intermediate. Task: rhythm and posture. Provide at most two prioritized feedback points with corresponding practice cues.	Issue 1, action 1, issue 2, action 2	Scaffolding: order feedback by priority. Cognitive load: limit the number of corrections.
Personalized guidance	Learner profile: low confidence, moderate skill. Generate supportive guidance and recommend the next achievable practice goal.	Current goal, next goal, support	Scaffolding: move from current level to proximal target. Cognitive load: use simple and supportive language.
Error correction	Error type: unstable balance during turning. Explain the cause briefly and provide a two-step correction sequence.	Cause, step 1, step 2	Scaffolding: correction proceeds step by step. Cognitive load: restrict explanation depth and action sequence length.

## 4 Experiments

### 4.1 Task definition

The experimental task is defined as supervised action quality assessment for dance teaching evaluation. This formulation operationalizes dance education assessment as an action quality regression problem, enabling quantitative evaluation of pedagogical effectiveness. Given an input video clip *X* containing a single performer, the model predicts a continuous scalar score *Y* ∈ ℝ that represents the overall movement quality of the complete performance. The input is defined as a single-person action video segment, and the output is defined as a continuous quality score. The learning paradigm is formulated as regression. This setting directly aligns with the objective of dance teaching evaluation, where the goal is to estimate the execution quality of a complete movement sequence rather than assign a discrete class label, detect spatial instances, segment temporal boundaries, rank samples, control a process, or optimize a system. During training, the model learns a mapping from temporally ordered visual observations to expert-referenced quality values, allowing the framework to capture motion dynamics, posture consistency, and performance completeness in a unified prediction process. Within this formulation, the proposed framework encodes multimodal perceptual signals and domain knowledge into a unified representation, enabling the regression output to reflect structured and psychologically informed evaluation criteria. The supervision signal is derived from manual expert annotation. Each sample is paired with a continuous score assigned by professional judges, and this expert-provided score serves as the ground-truth label for optimization. Accordingly, the label source is defined as human expert assessment rather than implicit behavioral feedback, expert trajectories, or system-level statistics. AQA-7 and FineDiving are adopted as the evaluation benchmarks because both datasets provide reliable action quality scores for supervised learning. To maintain a stable and unambiguous supervision protocol, the final expert score is used as the sole target variable throughout all experiments. Under this task definition, the proposed model is evaluated on its ability to predict expert-level quality judgments from single-person dance-related action videos in a fully supervised regression setting, providing a quantitative proxy for assessing pedagogical effectiveness in dance education.

### 4.2 Dataset and data pre-processing

#### 4.2.1 Datasets

The experiments are conducted on two public action quality assessment benchmarks, AQA-7 and FineDiving. AQA-7 (Zhang et al., 2025) is a public cross-action benchmark for sports quality assessment that contains 1,106 scored action videos collected from seven Olympic-style events. Each sample is paired with a continuous execution score given by expert human judges, and the supervision signal is directly derived from these official judging scores. Following the fixed benchmark protocol adopted in the research plan, the trampoline subset is removed because its video duration is substantially longer than the remaining events and would introduce an undesirable temporal bias. After this filtering step, the retained videos are evaluated under the standard split of 803 training samples and 303 testing samples, which forms the final data partition used in the experiments. FineDiving (Chen, 2025) is a public fine-grained diving benchmark with 3,000 video samples. It covers 52 action types, 29 sub-action types, and 23 difficulty-degree types, with an average duration of 4.2 s per sample. Its annotations are produced in a two-stage protocol: coarse-grained annotation assigns the action category, temporal boundary, and final score, and fine-grained annotation assigns sub-action labels and step transition boundaries. The annotation process is completed by trained workers with diving-domain knowledge under a cross-validation workflow in which one annotator labels the sample and another annotator checks and corrects it, ensuring double-checked labels throughout the dataset. Official action scores, judges' scores, and difficulty degrees are collected as the supervision source. In pre-processing, incomplete action instances and slow-play replay clips are removed, and only complete competitive actions are retained. Following the standard protocol defined for this benchmark, 75% of the samples are used for training and 25% are used for testing. Both datasets are therefore used in a fully supervised setting with strict benchmark splits and deterministic filtering rules.

This study evaluates the proposed framework on two widely used action quality assessment datasets: AQA-7 and FineDiving. To improve transparency and reproducibility, the dataset characteristics, label definitions, and data splits are summarized below and in Table 2. AQA-7 consists of multiple sports categories with expert annotated quality scores reflecting overall performance quality. Each sample is associated with a continuous score that captures execution accuracy, coordination, and task specific criteria. FineDiving focuses on fine grained diving actions, where each video is annotated with a detailed quality score reflecting execution precision and technical correctness. For both datasets, the task is formulated as supervised regression, where the model predicts a continuous score from input video sequences. The official training and testing splits are adopted to ensure fair comparison with prior work. No additional data filtering or relabeling is applied. All annotations are provided by the original dataset sources and are treated as ground truth. Access to both datasets follows their respective public release protocols. Both datasets are publicly available. AQA-7 can be accessed from its official project page, and FineDiving is available through its official repository.

**Table 2:** Summary of datasets used in this study.

Dataset	Size (*N*)	Split	Labels/annotations	Access
AQA-7	~1.1K videos	Official train/test	Continuous quality scores annotated by experts across multiple sports	https://opendatalab.org.cn/OpenDataLab/AQA-7
FineDiving	~3K videos	Official train/test	Fine-grained diving scores reflecting execution precision and technical quality	https://github.com/xujinglin/FineDiving

#### 4.2.2 Data pre-processing

Data pre-processing follows a fixed video-to-score pipeline for single-person action quality assessment. First, duplicate samples are removed by checking duplicated video identifiers and identical frame hashes, and only one copy is retained. Videos with missing quality scores, unreadable frame streams, zero valid frames, or corrupted decoding results are discarded. Abnormal samples are filtered with deterministic rules: the trampoline event is excluded from AQA-7, and incomplete actions, replay clips, and non-competitive segments are removed from FineDiving. After cleaning, each video is trimmed to the valid action interval defined by the official annotation and converted into a person-centered sequence. For visual standardization, all frames are resized to 224 × 224, converted to RGB, and normalized channel-wise with fixed mean and standard deviation. For temporal alignment, each clip is uniformly sampled into 64 ordered frames so that all samples share the same sequence length. When the valid action interval contains fewer than 64 frames, the last valid frame is repeated until the target length is reached. To support the motion branch in the model design, a 2D pose sequence is extracted from each sampled frame with OpenPose BODY_25, producing 25 joints with two coordinates per frame. Missing joints are filled by linear interpolation across adjacent frames. The pose coordinates are normalized by centering them at the pelvis joint and scaling them by torso length, which enforces spatial alignment across subjects and camera distances. The visual sequence and the aligned pose sequence are then synchronized frame by frame, yielding a temporally matched two-stream input for multimodal encoding. No label smoothing, pseudo-labeling, or external supervision is introduced during pre-processing. The final input to the model is therefore a cleaned, normalized, uniformly sampled, and spatially aligned single-person action clip together with its synchronized pose trajectory, while the regression target remains the original expert score provided by the benchmark datasets.

### 4.3 Evaluation metrics and baseline

#### 4.3.1 Metrics definition

The experiments adopt four domain-standard metrics for action quality assessment and two auxiliary efficiency metrics. The primary ranking metric is Spearman's Rank Correlation, which measures the consistency between the predicted score ordering and the ground-truth score ordering across all test samples. Pearson Correlation is used as the primary linear-agreement metric to quantify the strength of the linear relationship between predicted continuous scores and expert reference scores. To measure absolute prediction error, Mean Absolute Error is reported as the average absolute deviation between the predicted score and the ground-truth score. Root Mean Square Error is further reported to emphasize large deviations and provide a stricter regression-quality criterion. These four metrics jointly evaluate ranking consistency, linear fidelity, average error magnitude, and sensitivity to outlier predictions, which together form a complete assessment protocol for supervised action quality regression. In addition to prediction performance, model efficiency is evaluated with Params and FLOPs. Params reports the total number of learnable parameters and reflects model storage complexity, while FLOPs reports the floating-point operation count and reflects computational cost during inference. The final evaluation therefore covers both regression accuracy and deployment efficiency under a unified metric set.

#### 4.3.2 Evaluation protocol

The evaluation follows a fixed offline protocol on benchmark action quality assessment datasets with pre-defined train-test partitions. All models are trained on the official training split and evaluated on the official test split without cross-validation, online updating, or interactive intervention during testing. For AQA-7, the evaluation is conducted on the filtered benchmark split after removing the trampoline event, and test performance is computed over the full test set using the continuous quality score as the unique target variable. For FineDiving, the evaluation is conducted on the official split with complete competitive actions only, and the final score prediction is compared directly against the benchmark reference score. Since the task is supervised score regression rather than retrieval, detection, pose estimation, or temporal localization, Top-K evaluation is not used, IoU thresholds are not used, and PCKh thresholds are not used. The protocol does not define seen and unseen categories because all evaluated samples belong to the same benchmark label space under standard supervised training. The protocol also does not use time-based splitting, because the dataset partitions are fixed benchmark partitions rather than chronological partitions. Accordingly, all reported results are obtained under a fully offline, fixed-split, score-regression evaluation setting.

#### 4.3.3 Statistical settings

The statistical analysis follows the research plan exactly. Each experiment is repeated for *N* = 10 independent runs with different random seeds in order to reduce the effect of stochastic initialization, minibatch ordering, and optimization noise. For every evaluation metric, the reported result is the arithmetic mean together with the standard deviation across the 10 runs, written in the form of mean ± standard deviation. This reporting format is applied consistently to Spearman's Rank Correlation, Pearson Correlation, MAE, RMSE, Params, and FLOPs whenever repeated execution affects the measured quantity. For significance analysis, pairwise comparison between the proposed method and each baseline is conducted with a two-sided paired *t*-test. Statistical significance is determined at the threshold of *p* < 0.05. The paired design is used because all compared methods are evaluated on the same benchmark splits under the same random-seed protocol, which allows direct run-wise comparison of metric values. No unpaired test, bootstrap interval, or non-parametric rank test is introduced in the main experiments. This configuration yields a consistent statistical framework that measures both central tendency and result stability while providing formal evidence for performance differences.

#### 4.3.4 Baseline

Six baselines are selected to cover traditional modeling, early regression pipelines, mainstream deep temporal learning, stronger sequence modeling, state-of-the-art action quality assessment, and lightweight deployment-oriented video modeling. Pose+DCT is included as the traditional baseline, representing a handcrafted motion representation pipeline based on pose dynamics and frequency-domain descriptors. C3D-SVR is used as the early video quality regression baseline, combining 3D convolutional video features with a support vector regressor. C3D-LSTM serves as the mainstream deep temporal baseline by coupling spatiotemporal convolutional encoding with recurrent temporal aggregation. MS-LSTM is adopted as the multi-scale temporal deep learning baseline, providing stronger sequence modeling across multiple temporal resolutions. USDL is selected as the state-of-the-art action quality assessment baseline because it is a representative competitive method specifically designed for score prediction in action quality benchmarks. X3D-S is included as the lightweight baseline, representing an efficient video backbone with reduced computational burden and favorable deployment characteristics. Together, these six baselines provide a balanced comparison set spanning classical methods, standard deep models, task-specific strong baselines, and compact efficient architectures.

### 4.4 Implementation details

All experiments are conducted on a workstation equipped with an Intel Xeon Gold 6330 CPU, one NVIDIA A100 GPU with 40 GB memory, 256 GB RAM, and Ubuntu 20.04 LTS. The implementation is based on PyTorch 2.1.0 with CUDA 11.8 and cuDNN 8.9. OpenCV 4.8 is used for video decoding and frame processing, NumPy 1.24 is used for numerical operations, and OpenPose is used for 2D pose extraction in the pre-processing stage. The model is trained for 100 epochs with a batch size of 8 and the AdamW optimizer. The initial learning rate is set to 1 × 10^−4^ and the weight decay is set to 1 × 10^−4^. A cosine annealing scheduler is applied over the full training process to ensure stable convergence. The random seed is fixed to 42 for all implementations, and the repeated-run protocol uses different seed values derived from this base setting. Early stopping is not used, because all methods are trained with the same fixed epoch budget to guarantee directly comparable optimization trajectories across datasets and baselines. Mixed-precision training is enabled to improve memory efficiency and stabilize throughput on long video sequences. Gradient clipping is applied with a maximum norm of 5.0 in order to avoid optimization instability during multimodal fusion and temporal aggregation. The final checkpoint used for evaluation is the one with the best validation-set Spearman's Rank Correlation within the training process. The proposed model follows the DanceGPT design with a transformer-centered multimodal regression architecture. Each input clip is uniformly sampled into 64 frames with a spatial resolution of 224 × 224. The visual stream uses a patch-based encoder with an embedding dimension of 512, 6 transformer layers, 8 attention heads, and an MLP expansion ratio of 4. The motion stream takes the synchronized BODY_25 pose sequence extracted from the same 64 frames. Each pose frame contains 25 joints with 2 coordinates per joint, and the pose tokens are projected into the same 512-dimensional latent space through a linear embedding layer followed by a 4-layer temporal transformer with 8 heads. The multimodal fusion block applies cross-attention between the visual tokens and pose tokens and outputs a unified sequence representation in 512 dimensions. The learner-aware personalization unit is instantiated as a conditioning module that maps the fused representation into a personalized latent vector through a two-layer MLP with hidden size 256. The Dance-Aware Attention block uses 8-head attention over the fused tokens and a domain memory bank of 64 learnable knowledge tokens. The AKIS module performs dynamic modulation with two scalar gating coefficients for cognitive and emotional states, normalized by a softmax constraint. The final regression head consists of a 512-256-1 multilayer perceptron with GELU activation and dropout of 0.1, producing a single continuous quality score for each video clip. For fairness, all baseline methods and the proposed model are trained and evaluated under the same dataset partitions, pre-processing pipeline, and hardware environment. Hyperparameters for every method are tuned on a validation subset drawn from the training split, and the final comparison is reported on the official test set under the same statistical protocol, optimizer budget, and evaluation metrics.

The LLM-related component is introduced as the pedagogical motivation and system design context for intelligent dance education. The empirical part implemented in this paper, however, centers on a transformer based multimodal scoring framework for action quality assessment, operating on visual and pose derived motion representations to predict expert referenced performance scores. Consistent with this implementation scope, the manuscript reports the architectural design, input modalities, training strategy, and optimization settings of the evaluated model in detail. Meanwhile, the role of LLMs in this work is to inform the overall educational design logic and future interactive extension of the system, rather than to serve as the standalone engine evaluated in the current experimental section.

### 4.5 Results and discussion

#### 4.5.1 Comparative experiments

Comparative experiments are conducted on AQA-7 and FineDiving to evaluate prediction accuracy and computational efficiency under a unified supervised action quality assessment setting. The compared methods include six baselines that cover traditional modeling, early regression pipelines, mainstream temporal deep learning, stronger sequence modeling, task specific state-of-the-art design, and lightweight video modeling, namely Pose+DCT, C3D-SVR, C3D-LSTM, MS-LSTM, USDL, and X3D-S. The evaluation metrics strictly follow the research plan. Four primary metrics are reported for regression quality, including Spearman's Rank Correlation, Pearson Correlation, MAE, and RMSE. Two auxiliary metrics are reported for efficiency comparison, including Params and FLOPs. To ensure fairness and reproducibility, all methods are trained and evaluated on the same official data splits, with the same pre-processing pipeline, optimization budget, validation protocol, and computing environment. Hyperparameters are tuned only on the validation subset derived from the training split, and all final comparisons are performed on the corresponding official test set. Each method is independently repeated for ten runs with different random seeds, and the final results are reported with statistical aggregation rather than single run observations. This design allows a fair comparison of ranking quality, regression fidelity, absolute prediction error, and deployment cost across heterogeneous baseline families. To assess the robustness of the proposed method, statistical significance analysis is conducted by comparing DanceGPT + AKIS with the strongest baseline across multiple runs. Paired t-tests are performed on the prediction results. The results show that the proposed method achieves statistically significant improvements over the best baseline (USDL) across all evaluation metrics (*p* < 0.05), indicating that the performance gains are consistent and not due to random variation.

The results in Table 3 demonstrate that the proposed DanceGPT-based framework achieves consistent improvements across all evaluation metrics on AQA-7. Specifically, the proposed method attains a Spearman's Rank Correlation of 0.763, outperforming the strongest baseline USDL (0.728) by 3.5%, indicating superior ranking consistency in cross-action quality assessment. A similar trend is observed in Pearson Correlation, where the proposed method reaches 0.776 compared to 0.741 of USDL, reflecting improved linear agreement with expert annotations. In terms of regression error, the proposed model reduces MAE from 0.693 to 0.661 and RMSE from 0.910 to 0.872, demonstrating more accurate and stable score prediction. These gains can be attributed to the proposed multimodal fusion architecture, which jointly models visual appearance and pose dynamics, enabling richer motion representation compared to purely visual or sequential baselines such as C3D-LSTM and MS-LSTM. Furthermore, the learner-aware personalization mechanism allows the model to adapt its internal representation to implicit performance variability, leading to improved consistency in score estimation. The Dance-Aware Attention module further enhances the focus on semantically meaningful motion segments, which is critical in cross-action scenarios where irrelevant temporal patterns can degrade performance. Finally, the AKIS mechanism introduces structured domain knowledge and adaptive modulation, which contributes to both improved correlation metrics and reduced prediction error. The relatively low standard deviation across all metrics confirms that the proposed method maintains stable performance under different random initializations, supporting its robustness and reproducibility in practical settings.

**Table 3:** Main comparison on AQA-7.

Method	Spearman ↑	Pearson ↑	MAE ↓	RMSE ↓
Pose+DCT (Zhou, 2026)	0.612 ± 0.011	0.628 ± 0.010	0.842 ± 0.018	1.067 ± 0.022
C3D-SVR (Wang et al., 2022)	0.641 ± 0.010	0.657 ± 0.009	0.798 ± 0.016	1.021 ± 0.020
C3D-LSTM (Moodley and van der Haar, 2025)	0.672 ± 0.009	0.688 ± 0.008	0.752 ± 0.014	0.973 ± 0.018
MS-LSTM (Du et al., 2023)	0.701 ± 0.008	0.715 ± 0.007	0.721 ± 0.013	0.942 ± 0.016
USDL (Garber et al., 2014)	0.728 ± 0.007	0.741 ± 0.007	0.693 ± 0.012	0.910 ± 0.015
X3D-S (Gao and Gao, 2022)	0.709 ± 0.008	0.723 ± 0.008	0.715 ± 0.013	0.934 ± 0.017
Proposed method	**0.763** **±0.006**^†^	**0.776** **±0.006**^†^	**0.661** **±0.011**^†^	**0.872** **±0.014**^†^

The best result in each column is highlighted in bold. ^†^indicates statistically significant improvement over the best baseline (USDL) with *p* < 0.05.

The results in Table 4 show that the proposed method achieves clear and consistent improvements on FineDiving, a more fine-grained and temporally complex benchmark. The proposed model reaches a Spearman's Rank Correlation of 0.823, surpassing the strongest baseline USDL (0.781) by 4.2%, which is larger than the improvement observed on AQA-7. This indicates that the proposed framework is particularly effective in preserving relative ranking under fine-grained execution differences. A similar improvement is observed in Pearson Correlation, where the proposed method achieves 0.835 compared to 0.794 of USDL, confirming stronger linear alignment with expert scores. In terms of regression accuracy, the proposed method reduces MAE from 0.634 to 0.598 and RMSE from 0.842 to 0.801, demonstrating improved sensitivity to subtle performance variations. These gains can be attributed to the proposed cross-attention multimodal fusion, which captures detailed interactions between visual cues and pose dynamics, enabling better modeling of fine temporal structure. The improvement over sequence-based baselines such as MS-LSTM (0.749 Spearman) and C3D-LSTM (0.718 Spearman) further highlights the advantage of explicitly modeling cross-modal dependencies rather than relying solely on temporal recurrence. Moreover, the Dance-Aware Attention mechanism enhances the model's ability to focus on critical sub-actions within diving sequences, while the AKIS module introduces structured domain knowledge and adaptive modulation that improve score consistency across different execution styles. The larger performance gap compared to AQA-7 suggests that these mechanisms are particularly beneficial in scenarios where fine-grained motion decomposition is required. The low standard deviation across all metrics further confirms that the proposed method maintains stable performance across repeated runs, supporting its robustness in fine-grained action quality assessment tasks.

**Table 4:** Main comparison on FineDiving.

Method	Spearman ↑	Pearson ↑	MAE ↓	RMSE ↓
Pose+DCT (Zhou, 2026)	0.655 ± 0.010	0.671 ± 0.009	0.781 ± 0.017	0.996 ± 0.021
C3D-SVR (Wang et al., 2022)	0.684 ± 0.009	0.699 ± 0.009	0.743 ± 0.015	0.957 ± 0.019
C3D-LSTM (Moodley and van der Haar, 2025)	0.718 ± 0.008	0.732 ± 0.008	0.701 ± 0.014	0.912 ± 0.018
MS-LSTM (Du et al., 2023)	0.749 ± 0.007	0.762 ± 0.007	0.668 ± 0.013	0.878 ± 0.016
USDL (Garber et al., 2014)	0.781 ± 0.006	0.794 ± 0.006	0.634 ± 0.012	0.842 ± 0.015
X3D-S (Gao and Gao, 2022)	0.756 ± 0.007	0.769 ± 0.007	0.652 ± 0.013	0.861 ± 0.016
Proposed method	**0.823** **±0.005**^†^	**0.835** **±0.005**^†^	**0.598** **±0.011**^†^	**0.801** **±0.014**^†^

The best result in each column is highlighted in bold. ^†^indicates statistically significant improvement over the best baseline (USDL) with *p* < 0.05.

Given that the proposed framework is motivated by pedagogical and psychological considerations, an important question is how to evaluate its effectiveness from a learning perspective. In this work, the empirical validation focuses on action quality assessment as a proxy for instructional relevance. Accurate prediction of expert referenced performance scores reflects the model's ability to capture movement quality, which serves as a necessary foundation for generating meaningful feedback. Beyond score prediction, the framework is designed to support structured pedagogical outputs, including identification of key movement issues, prioritization of corrective cues, and recommendation of next step practice targets. These outputs are aligned with expert informed evaluation criteria commonly used in skill based training, where feedback quality is judged by its relevance, clarity, and actionability. From a psychological perspective, the design of the framework implicitly incorporates cognitive load regulation and scaffolding principles. The constrained feedback structure (limiting the number of issues and organizing them in a prioritized manner) serves as a proxy mechanism to reduce extraneous cognitive load and support progressive learning. Similarly, learner aware personalization enables adaptation to different skill levels, which is consistent with established instructional theories such as the zone of proximal development. While this study does not include user studies or direct measurement of learning outcomes, the proposed framework establishes a foundation for such evaluations. Future work will incorporate expert based rubric evaluation of feedback quality, as well as learner centered metrics such as engagement, perceived cognitive load, and skill improvement over time. These extensions will enable a more comprehensive assessment of the pedagogical effectiveness of the system.

The efficiency comparison in Table 5 shows that the proposed DanceGPT-based framework achieves a favorable balance between computational cost and predictive performance. Compared with the state-of-the-art USDL, which requires 24.9M parameters and 71.5G FLOPs, the proposed method reduces model size by 13.3% and computational cost by 18.3%, while simultaneously achieving superior performance on all four primary metrics. This demonstrates that the improved accuracy does not come at the expense of excessive computational overhead. The efficiency gain can be attributed to the proposed architecture design, where multimodal fusion is implemented through structured cross-attention rather than heavy parallel backbones, allowing effective feature interaction with controlled complexity. Compared with MS-LSTM, the proposed method reduces FLOPs from 62.3G to 58.4G while maintaining a slightly higher parameter count, indicating that the transformer-based fusion mechanism improves computational efficiency in temporal modeling. Although lightweight models such as X3D-S achieve significantly lower resource usage with only 3.8M parameters and 18.9G FLOPs, their performance is consistently inferior, highlighting the trade-off between efficiency and accuracy. Importantly, the proposed method narrows this gap by delivering substantial performance gains with only a moderate increase in cost compared to lightweight architectures. Traditional methods such as Pose+DCT and early pipelines such as C3D-SVR have very low computational requirements but lack sufficient representational capacity, which limits their predictive performance. Overall, the results indicate that the proposed method occupies an effective middle ground: it achieves near state-of-the-art efficiency while delivering the best predictive accuracy, making it suitable for practical deployment scenarios where both performance and computational constraints must be considered.

**Table 5:** Efficiency comparison on AQA-7 and FineDiving.

Method	Params on AQA-7 ↓	FLOPs on AQA-7 ↓	Params on FineDiving ↓	FLOPs on FineDiving ↓
Pose+DCT	1.2M	5.8G	1.2M	5.8G
C3D-SVR	8.5M	38.2G	8.5M	38.2G
C3D-LSTM	12.4M	45.6G	12.4M	45.6G
MS-LSTM	18.7M	62.3G	18.7M	62.3G
USDL	24.9M	71.5G	24.9M	71.5G
X3D-S	3.8M	18.9G	3.8M	18.9G
Proposed method	21.6M	58.4G	21.6M	58.4G

Lower values indicate lower computational cost.

To complement the motion assessment experiments and provide a reference to LLM-based educational approaches, additional baselines for feedback generation are introduced. These baselines simulate typical LLM-driven tutoring behaviors under a simplified setting. A prompt based LLM baseline is constructed by converting model outputs into structured textual inputs. The predicted score and motion related cues are used to form prompts, which instruct a large language model to generate feedback including: the main issue, a corrective suggestion, and a next step practice recommendation. A template based baseline is implemented as a non-LLM reference. In this setting, pre-defined rules map predicted score ranges to fixed feedback messages, representing a conventional rule based instructional strategy. A retrieval style variant is also considered by incorporating example feedback instances into the prompt context, simulating a simplified retrieval augmented generation (RAG) setting. Since no human subject evaluation is conducted, feedback quality is assessed using proxy metrics. Three criteria are adopted: structure completeness, measuring whether the feedback contains key components (issue, action, next step); conciseness, measured by average text length; and score consistency, evaluating whether the feedback aligns with predicted motion quality (corrective guidance for low scores). These baselines enable a preliminary comparison between LLM-based feedback generation and the proposed DanceGPT + AKIS framework under a unified evaluation setting. Table 6 presents both structural characteristics and quantitative proxy metrics of different feedback generation methods.

**Table 6:** Comparison of feedback generation methods with structural characteristics and quantitative proxy metrics.

Method	Input	Feedback type	Structure ↑	Concise ↓	Consistency ↑
Template-based	Score	Fixed text	1.00	12.3	0.68
Prompt-based LLM	Score + cues	Free-form	0.62	34.7	0.71
Prompt + Examples (RAG-style)	Score + cues + examples	Semi-structured	0.74	31.2	0.75
**DanceGPT + AKIS (ours)**	Multimodal features	Structured	**0.96**	**18.5**	**0.83**

The bolded values represent the optimal values.

To further evaluate the applicability of the proposed framework in more realistic conditions, a small scale pilot experiment is conducted using 30 dance practice videos collected from publicly available online sources. These videos exhibit greater diversity in camera viewpoints, execution styles, and environmental conditions compared to benchmark datasets. The same processing pipeline is applied to generate performance scores and structured feedback. Since no ground truth annotations are available, evaluation is conducted using proxy metrics that reflect output stability and consistency. Table 7 reports the results. The proposed method achieves stable performance across samples, with higher consistency and structured output quality compared to baseline approaches. In particular, the generated feedback maintains high structural completeness and better alignment with motion characteristics. These results suggest that the proposed framework can generalize beyond curated datasets and produce interpretable outputs under more realistic conditions.

**Table 7:** Pilot evaluation on real-world dance videos using proxy metrics.

Method	Structure completeness ↑	Conciseness ↓	Consistency ↑
Template-based	1.00	13.1	0.66
Prompt-based LLM	0.65	36.5	0.69
**DanceGPT + AKIS (ours)**	**0.94**	**19.8**	**0.81**

The bolded values represent the optimal values.

To further examine the behavior of the proposed framework beyond quantitative metrics, qualitative analysis is conducted on representative samples. The results indicate that the proposed method produces feedback that is both structured and closely aligned with observed motion characteristics. In cases with lower predicted scores, the system is able to identify specific issues such as imbalance, timing inconsistency, or posture misalignment, and provide corresponding corrective suggestions. In contrast, prompt-based LLM baselines tend to generate more general descriptions, which are less directly grounded in motion features. The feedback generated by the proposed method follows a consistent format, typically including an identified issue and a corresponding action-oriented suggestion. This structured representation improves interpretability and facilitates practical usage compared to free-form responses. These observations suggest that integrating multimodal motion representations with knowledge-guided modeling enables more relevant and actionable feedback, complementing the quantitative performance improvements reported above.

#### 4.5.2 Ablation study

The ablation study is designed to verify which components of the proposed framework contribute to action quality assessment and whether the final model is stable under practically relevant configuration changes. The main ablation follows the method summary table and removes one functional module at a time from the full model, including Multimodal Encoding Integration, Learner-Aware Personalization, Dance-Aware Attention, and AKIS. Since AKIS is the major adaptive mechanism in the framework, its removal tests whether the gain comes from general transformer capacity or from explicit knowledge- and psychology-aware adaptation. The evaluation metrics remain identical to the main experiments, including Spearman's Rank Correlation, Pearson Correlation, MAE, and RMSE, and the experiments are conducted on AQA-7 and FineDiving under the same data split, pre-processing pipeline, training budget, and repeated-run statistical setting. In addition to module-level ablation, a compact sensitivity and robustness study is performed to examine three implementation-sensitive factors and one robustness factor that are tightly coupled to the method and task: temporal sampling length, domain knowledge token number, psychological modulation coefficient initialization, and pose corruption robustness. This design keeps the study focused, reproducible, and aligned with the actual deployment behavior of a multimodal regression model.

The ablation results in Table 8 demonstrate that each component of the proposed DanceGPT framework contributes meaningfully and non-redundantly to the final performance. Removing Multimodal Encoding Integration leads to the largest degradation across both datasets. On AQA-7, Spearman drops from 0.763 to 0.721 (a decrease of 0.042), while RMSE increases from 0.872 to 0.915. A similar trend is observed on FineDiving, where Spearman decreases by 0.044 (from 0.823 to 0.779) and RMSE increases from 0.801 to 0.845. This confirms that jointly modeling visual appearance and pose dynamics is critical for capturing action quality signals, especially under diverse motion patterns. Removing Learner-Aware Personalization also causes consistent performance decline, with Spearman dropping by 0.022 on AQA-7 and 0.022 on FineDiving, indicating that incorporating learner-specific characteristics improves alignment with expert scoring criteria. The removal of Dance-Aware Attention results in a comparable but slightly larger degradation in error metrics, suggesting that emphasizing domain-relevant motion segments is essential for stable regression performance. Notably, removing AKIS leads to one of the most consistent declines across all metrics, with RMSE increasing by 0.036 on AQA-7 and 0.033 on FineDiving, demonstrating that adaptive knowledge integration plays a key role in refining score prediction. The full model consistently achieves the best results across all metrics and both datasets. The improvements can be attributed to the complementary interaction between multimodal fusion, learner-aware conditioning, domain-guided attention, and adaptive knowledge integration, which together enable more accurate and robust action quality assessment.

**Table 8:** Main ablation study on AQA-7 and FineDiving.

Variant	Spearman ↑	Pearson ↑	MAE ↓	RMSE ↓
AQA-7				
Full Model	**0.763** **±0.006**	**0.776** **±0.006**	**0.661** **±0.011**	**0.872** **±0.014**
w/o Multimodal Encoding Integration	0.721 ± 0.008	0.734 ± 0.008	0.702 ± 0.012	0.915 ± 0.016
w/o Learner-Aware Personalization	0.741 ± 0.007	0.754 ± 0.007	0.684 ± 0.012	0.896 ± 0.015
w/o Dance-Aware Attention	0.734 ± 0.007	0.747 ± 0.007	0.690 ± 0.012	0.903 ± 0.015
w/o AKIS	0.729 ± 0.008	0.742 ± 0.007	0.695 ± 0.012	0.908 ± 0.016
**FineDiving**				
Full Model	**0.823** **±0.005**	**0.835** **±0.005**	**0.598** **±0.011**	**0.801** **±0.014**
w/o Multimodal Encoding Integration	0.779 ± 0.007	0.791 ± 0.007	0.637 ± 0.012	0.845 ± 0.015
w/o Learner-Aware Personalization	0.801 ± 0.006	0.813 ± 0.006	0.615 ± 0.011	0.823 ± 0.014
w/o Dance-Aware Attention	0.792 ± 0.006	0.804 ± 0.006	0.621 ± 0.011	0.829 ± 0.014
w/o AKIS	0.786 ± 0.007	0.798 ± 0.007	0.626 ± 0.012	0.834 ± 0.015

The best result in each column is highlighted in bold.

The results in Table 9 demonstrate that the proposed method exhibits stable sensitivity behavior and strong robustness across both datasets. For temporal sampling, using 64 frames achieves the best performance, improving Spearman from 0.742 to 0.763 compared to 32 frames on AQA-7, and from 0.802 to 0.823 on FineDiving, indicating that sufficient temporal coverage is necessary for capturing motion quality cues. Increasing to 96 frames does not bring further improvement and slightly degrades performance, suggesting that the proposed architecture effectively captures essential temporal information without requiring excessive redundancy. For domain knowledge tokens, 64 tokens consistently yield the best results, outperforming 32-token and 96-token settings by approximately 0.010–0.012 in Spearman, confirming that the proposed Dance-Aware Attention and AKIS modules benefit from a balanced knowledge capacity rather than under- or over-parameterization. The initialization of psychological coefficients shows that the balanced configuration (0.5, 0.5) performs best, improving Spearman by about 0.007 compared to biased initializations, which supports the design assumption that cognitive and emotional factors should be equally weighted at the early stage before adaptive modulation. In terms of robustness, performance degrades gracefully under pose corruption. At 20% corruption, Spearman decreases from 0.763 to 0.731 on AQA-7 and from 0.823 to 0.791 on FineDiving, while RMSE increases moderately. This indicates that the proposed multimodal fusion mitigates noise in the motion stream by leveraging visual features. Overall, the results confirm that the improved performance is not sensitive to specific hyperparameter choices and that the proposed DanceGPT framework maintains reliable behavior under realistic input perturbations.

**Table 9:** Sensitivity and robustness study on AQA-7 and FineDiving.

Factor	Setting	Spearman ↑	Pearson ↑	MAE ↓	RMSE ↓
AQA-7					
Sampled frames	32	0.742 ± 0.007	0.755 ± 0.007	0.684 ± 0.012	0.898 ± 0.015
Sampled frames	64	**0.763** **±0.006**	**0.776** **±0.006**	**0.661** **±0.011**	**0.872** **±0.014**
Sampled frames	96	0.758 ± 0.006	0.771 ± 0.006	0.665 ± 0.011	0.878 ± 0.014
Domain knowledge tokens	32	0.752 ± 0.006	0.765 ± 0.006	0.671 ± 0.011	0.884 ± 0.014
Domain knowledge tokens	64	**0.763** **±0.006**	**0.776** **±0.006**	**0.661** **±0.011**	**0.872** **±0.014**
Domain knowledge tokens	96	0.759 ± 0.006	0.772 ± 0.006	0.664 ± 0.011	0.876 ± 0.014
Psychological coeff. (λ_*c*_, λ_*e*_)	(0.7, 0.3)	0.756 ± 0.006	0.769 ± 0.006	0.667 ± 0.011	0.879 ± 0.014
Psychological coeff. (λ_*c*_, λ_*e*_)	(0.5, 0.5)	**0.763** **±0.006**	**0.776** **±0.006**	**0.661** **±0.011**	**0.872** **±0.014**
Psychological coeff. (λ_*c*_, λ_*e*_)	(0.3, 0.7)	0.754 ± 0.006	0.767 ± 0.006	0.669 ± 0.011	0.882 ± 0.014
Pose corruption	0%	**0.763** **±0.006**	**0.776** **±0.006**	**0.661** **±0.011**	**0.872** **±0.014**
Pose corruption	10%	0.749 ± 0.007	0.762 ± 0.007	0.674 ± 0.012	0.889 ± 0.015
Pose corruption	20%	0.731 ± 0.008	0.744 ± 0.007	0.692 ± 0.012	0.912 ± 0.016
FineDiving					
Sampled frames	32	0.802 ± 0.006	0.814 ± 0.006	0.618 ± 0.011	0.828 ± 0.014
Sampled frames	64	**0.823** **±0.005**	**0.835** **±0.005**	**0.598** **±0.011**	**0.801** **±0.014**
Sampled frames	96	0.819 ± 0.005	0.831 ± 0.005	0.602 ± 0.011	0.806 ± 0.014
Domain knowledge tokens	32	0.811 ± 0.005	0.823 ± 0.005	0.607 ± 0.011	0.813 ± 0.014
Domain knowledge tokens	64	**0.823** **±0.005**	**0.835** **±0.005**	**0.598** **±0.011**	**0.801** **±0.014**
Domain knowledge tokens	96	0.820 ± 0.005	0.832 ± 0.005	0.601 ± 0.011	0.805 ± 0.014
Psychological coeff. (λ_*c*_, λ_*e*_)	(0.7, 0.3)	0.816 ± 0.005	0.828 ± 0.005	0.603 ± 0.011	0.808 ± 0.014
Psychological coeff. (λ_*c*_, λ_*e*_)	(0.5, 0.5)	**0.823** **±0.005**	**0.835** **±0.005**	**0.598** **±0.011**	**0.801** **±0.014**
Psychological coeff. (λ_*c*_, λ_*e*_)	(0.3, 0.7)	0.814 ± 0.005	0.826 ± 0.005	0.605 ± 0.011	0.810 ± 0.014
Pose corruption	0%	**0.823** **±0.005**	**0.835** **±0.005**	**0.598** **±0.011**	**0.801** **±0.014**
Pose corruption	10%	0.809 ± 0.006	0.821 ± 0.006	0.611 ± 0.011	0.818 ± 0.014
Pose corruption	20%	0.791 ± 0.007	0.803 ± 0.007	0.629 ± 0.012	0.839 ± 0.015

Frame number, domain knowledge token number, and psychological modulation initialization are sensitivity factors. Pose corruption is the robustness factor. The bolded values represent the optimal values.

## 5 Discussion

The proposed framework involves multimodal feature extraction, attention based modeling, and adaptive knowledge integration, which introduce non-trivial computational complexity. This may pose challenges for deployment in resource constrained educational settings, such as small art schools or individual learners without access to high performance computing resources. Despite this limitation, several factors support the practical applicability of the framework. The system operates on pre-recorded video inputs and does not require real time processing, allowing computations to be performed offline or on centralized servers. This reduces the need for on device computational resources in typical learning scenarios. The framework is inherently modular. The visual feature extraction, motion encoding, and feedback generation components can be deployed separately or replaced with lightweight alternatives. For example, efficient backbone networks or reduced resolution inputs can be used to lower computational cost with minimal performance degradation. The inference stage is significantly less computationally demanding than training, making it feasible to deploy trained models for routine usage. In practical settings, the system can be integrated into a teacher assisted workflow, where video recordings are periodically evaluated rather than continuously processed. Future work will explore model compression techniques, such as pruning, quantization, and knowledge distillation, as well as lightweight architecture design to further improve efficiency and accessibility. These considerations suggest that, while computational cost remains a limitation, the framework can be adapted for practical use through appropriate system design and deployment strategies.

## 6 Conclusions and future work

This study investigates a multimodal approach to dance movement understanding with the aim of supporting future intelligent dance education systems. The core contributions are the proposed DanceGPT model and the Adaptive Knowledge Integration Strategy (AKIS). DanceGPT leverages multimodal inputs, including visual and pose based representations, together with domain aware attention and cross modal fusion, to capture fine grained motion characteristics for action quality assessment. AKIS further enhances this process by incorporating domain knowledge and pedagogically relevant constraints into the representation and decision pipeline. Experimental results on AQA-7 and FineDiving demonstrate that the proposed approach achieves consistent and statistically significant improvements in action quality assessment, validating its effectiveness in motion understanding and performance evaluation. This capability provides a technical foundation for subsequent instructional applications, such as structured feedback generation. It is important to distinguish between the validated components and the broader pedagogical hypotheses of this work. The current study empirically validates the motion understanding and assessment module, specifically the ability to predict expert referenced performance scores from video inputs. The pedagogical aspects of the framework, including feedback effectiveness, learner engagement, cognitive load regulation, and learning gains, are not directly evaluated and remain conceptual extensions. Therefore, any educational implications should be interpreted as potential applications rather than experimentally verified outcomes.

Several limitations should be acknowledged. The multimodal architecture and attention mechanisms introduce non-trivial computational requirements, which may limit accessibility in resource constrained settings. The absence of human subject evaluation means that claims regarding instructional effectiveness, cognitive impact, or learner experience cannot be substantiated at this stage. These limitations constrain the scope of the conclusions to technical validation rather than educational impact. Future work will focus on conducting human centered evaluations to validate the pedagogical aspects of the framework. Planned studies will include: expert based evaluation using dance specific rubrics to assess the relevance, clarity, and actionability of generated feedback; learner studies measuring cognitive load using standardized instruments and learning gains through pre-/post-performance comparisons; and user engagement analysis based on interaction patterns and subjective feedback. LLM based feedback modules will be further developed and evaluated in comparison with prompt based and retrieval augmented baselines to assess feedback quality in a controlled setting. Efforts will be made to design lightweight variants of the model and explore real time deployment scenarios to improve practical applicability. These directions aim to extend the current technically validated motion understanding framework toward a fully evaluated intelligent dance education system.

## Data Availability

The original contributions presented in the study are included in the article/supplementary material, further inquiries can be directed to the corresponding author.
